# The first report of Japanese antimicrobial use measured by national database based on health insurance claims data (2011–2013): comparison with sales data, and trend analysis stratified by antimicrobial category and age group

**DOI:** 10.1007/s15010-017-1097-x

**Published:** 2017-12-22

**Authors:** Daisuke Yamasaki, Masaki Tanabe, Yuichi Muraki, Genta Kato, Norio Ohmagari, Tetsuya Yagi

**Affiliations:** 1 Department of Infection Control and Prevention, Mie University Hospital, 2-174 Edobashi, Tsu, Mie 514-8507 Japan; 2 Department of Clinical Pharmacoepidemiology, Kyoto Pharmaceutical University, Kyoto, Kyoto Japan; 3 Solutions Center for Health Insurance Claims, Kyoto University Hospital, Kyoto, Kyoto Japan; 4 Disease Control and Prevention Center, National Center for Global Health and Medicine, Shinjuku-ku, Tokyo, Japan; 5 Department of infectious Diseases, Nagoya University Hospital, Nagoya, Aichi Japan

**Keywords:** Antimicrobial use, Monitoring, National database, Japan

## Abstract

**Purpose:**

Our objective was to evaluate the utility of the national database (NDB) based on health insurance claims data for antimicrobial use (AMU) surveillance in medical institutions in Japan.

**Methods:**

The population-weighted total AMU expressed as defined daily doses (DDDs) per 1000 inhabitants per day (DID) was measured by the NDB. The data were compared with our previous study measured by the sales data. Trend analysis of DID from 2011 to 2013 and subgroup analysis stratified by antimicrobial category and age group were performed.

**Results:**

There was a significant linear correlation between the AMUs measured by the sales data and the NDB. Total oral and parenteral AMUs (expressed in DID) were 1.04-fold from 12.654 in 2011 to 13.202 in 2013 and 1.13-fold from 0.734 to 0.829, respectively. Percentage of oral form among total AMU was high with more than 94% during the study period. AMU in the children group (0–14 years) decreased from 2011 to 2013 regardless of dosage form, although the working age group (15–64 years) and elderly group (65 and above years) increased. Oral AMU in the working age group was approximately two-thirds of those in the other age groups. In contrast, parenteral AMU in the elderly group was extremely high compared to the other age groups.

**Conclusions:**

The trend of AMU stratified by antimicrobial category and age group were successfully measured using the NDB, which can be a tool to monitor outcome indices for the national action plan on antimicrobial resistance.

## Introduction

Antimicrobial resistance (AMR), resistance of a microorganism to an antimicrobial drug, is an increasingly serious problem globally and is recognized as one of the greatest potential threats to human health with serious consequences for public health [1, 2]. In May 2015, the World Health Assembly endorsed the Global Action Plan on Antimicrobial Resistance, and urged all Member States to develop relevant national action plans within 2 years [3]. In accordance with this demand, the Government of Japan developed a National Action Plan on Antimicrobial Resistance, presenting priorities to be implemented over the next 5 years to promote AMR measures in April 2016 [4].

Antimicrobial use (AMU) in medical institutions is closely associated with AMR, and AMU surveillance is one of several key actions to combat AMR [3–5]. The Japanese government set the national targets that reduced AMU in 2020 compared to 2013. However, nation-wide surveillance methods to monitor AMU in Japan have not been established yet. We previously reported AMU surveillance using pharmaceutical sales data through wholesalers [6]. This is the first and only report in Japan regarding the population-weighted consumption of oral and parenteral antimicrobials, expressed as defined daily dose (DDD) per 1000 inhabitants per day (DID). Sales data are a useful approach to monitor the trend of total AMU. However, detailed data regarding patients who received antimicrobials are not available. To promote appropriate antimicrobial use, age-specific quantities and patterns of AMU are needed.

Japan has a universal coverage of social health insurance, and people in Japan have an obligation to join it. A patient pays part of the cost on site and the rest is paid to each institution by insurers. At the end of the month, each medical facility sends a set of claims for reimbursement to insurers. For the production of a claim sheet of each patient for reimbursement, most medical facilities use a special computer system [7]. In 2008, Ministry of Health, Labour and Welfare (MHLW) started to construct the database of all electronic claim data, so-called national database of health insurance claims, and specific health checkups of Japan (NDB), which currently covers approximately 98% of healthcare services provided by health insurance [8]. The NDB has grown to become one of the largest databases in the world, and encompasses approximately 5 billion health insurance claims and 66 million health check and guidance data as of June 2012 [9, 10]. From 2011, MHLW opened this database to researchers, central and local governments for research and health policy discussion. NDB can be a powerful tool to survey the medical care situations in Japan. However, AMU surveillance using NDB has yet to be performed.

Accordingly, our objectives were to evaluate the utility of NDB for AMU surveillance in medical institutions, and to analyze the trend of quantities and patterns of AMU and its age-specific distributions.

## Methods

### Study design and data source

A retrospective nation-wide population-based descriptive study was conducted in Japan from January 1, 2011 to December 31, 2013. The quantities and patterns of total systemic antimicrobial prescription were analyzed using claim data in the NDB collected through the MHLW of Japan.

To use the database, our proposal document was reviewed and approved by the Advisory committee of the MHLW that composes of representatives of insurer and provider, persons from academics and journalist. AMU data prescribed at the hospital, clinic, and pharmacy through health insurance (medical and pharmaceutical claims) in 2011, 2012, and 2013 were extracted from NDB for analysis. Antiviral agents, antifungal agents, parasiticides, antituberculosis agents, and antileprosy agents were not included in this study.

The study was approved by the Institutional Ethical Committee of Mie University Graduate School of Medicine (no. 1587).

### Measures of AMU

Antimicrobial volume data extracted from NDB were converted to gram data. The data were standardized in accordance with the anatomical therapeutic chemical (ATC) classification using DDD as a measurement unit, as recommended by the World Health Organization Collaborating Centre for Drug Statistics Methodology [11]. The DDD is the assumed average maintenance dose per day for a drug used for its main indication in adults. The 2017 version of the ATC/DDD system was applied to all data. The daily dose of some antimicrobials for which DDDs were not defined in the ATC/DDD system was separately defined as JDDD using the approved maintenance dosages in Japan, as shown in our previous study [6]. The population-weighted total AMU was normalized as DDDs per 1000 inhabitants per day (DID) for multilateral analysis of the current trends on a national level as well as international comparison. Then, these DID values of each antimicrobial were integrated using the taxonomy of the ATC classification. Oral and parenteral antimicrobials were separately analyzed.

Trends of oral and parenteral antimicrobial DID in accordance with the ATC classification as calculated by the NDB were evaluated, and the DIDs by the NDB in 2011 and 2013 were directly compared with those calculated by sales data previously reported [6]. Thereafter, subgroup analysis stratified by each age class (in 5 year increments) and age group (0–14, 15–64, 65, and above years) in the inpatient and outpatient settings was performed. The data for Japan’s population in each age group were obtained from the results of a population survey report that was previously published by the Ministry of Internal Affairs and Communications of Japan.

### Statistical analysis

The statistical analyses were performed using IBM SPSS Statistics version 22.0 (IBM Corporation, Armonk, NY, USA). Pearson’s correlation analysis was used for relationship analysis. *P* < 0.05 was considered statistically significant.

## Results

Trends of oral and parenteral antimicrobial DID at the ATC 4th level from 2011 to 2013 are presented in Table 1. Although some antimicrobials showed a decrease, total oral and parenteral AMUs (expressed in DID) were increased by 1.04-fold from 12.654 in 2011 to 13.202 in 2013 and 1.13-fold from 0.734 to 0.829, respectively. The percentage of the oral antibiotics out of the total AMU was high, with more than 94%, during the study period.

**Table 1:** Trends of antimicrobial use stratified by ATC classification from 2011 to 2013

ATC 4th level	NDB				Sales data^b^	
	2011	2012	2013	Δ^a^	2011	2013
(a) Oral						
Teracycline (J01AA)	0.522	0.560	0.566	0.044	0.77	0.78
Amphenicols (J01BA)	0.000	0.000	0.000	0.000	0.00	0.00
Penicillins with extended spectrum (J01CA)	0.549	0.566	0.756	0.206	0.80	0.88
β-Lactamase-sensitive penicillins (J01CE)	0.024	0.024	0.021	− 0.003	0.01	0.01
Combinations of penicillins, including β-lactamase inhibitor (J01CR)	0.198	0.228	0.230	0.032	0.24	0.25
First-generation cephalosporins (J01DB)	0.113	0.106	0.104	− 0.009	0.08	0.07
Second-generation cephalosporins (J01DC)	0.061	0.058	0.056	− 0.005	0.33	0.30
Third-generation cephalosporins (J01DD)	2.960	3.016	2.930	− 0.030	3.57	3.47
Penems (J01DI)	0.097	0.104	0.102	0.005	0.13	0.13
Combinations of sulfonamides and trimethoprim, including derivatives (J01EE)	0.705	0.796	0.887	0.182	0.79	0.98
Short-acting macrolide (J01FA)	0.253	0.251	0.246	− 0.007	0.29	0.24
Intermediate-acting macrolide (J01FA)	3.941	4.011	3.995	0.054	4.00	3.84
Long-acting macrolide (J01FA)	0.704	0.679	0.577	− 0.127	0.89	0.76
Lincosamides (J01FF)	0.009	0.010	0.010	0.001	0.02	0.02
First-generation fluoroquinolones (J01MA)	0.033	0.033	0.031	− 0.002	0.04	0.03
Second-generation fluoroquinolones (J01MA)	1.778	1.913	1.837	0.059	1.93	1.91
Third-generation fluoroquinolones (J01MA)	0.598	0.713	0.739	0.141	0.66	0.82
Polymyxins (J01XB)	0.005	0.005	0.004	0.000	0.03	0.03
Other antibacterials (J01XX)	0.103	0.110	0.110	0.007	0.10	0.10
Total	12.654	13.183	13.202	0.548	14.66	14.61
(b) Parenteral						
Teracycline (J01AA)	0.012	0.012	0.011	− 0.001	0.004	0.004
Amphenicols (J01BA)	0.000	0.000	0.000	0.000	0.000	0.000
Penicillins with extended spectrum (J01CA)	0.025	0.026	0.028	0.003	0.025	0.027
β-Lactamase-sensitive penicillins (J01CE)	0.002	0.002	0.002	0.000	0.022	0.019
Combinations of penicillins, including β-lactamase inhibitor (J01CR)	0.185	0.202	0.227	0.042	0.316	0.389
First-generation cephalosporins (J01DB)	0.059	0.061	0.065	0.007	0.121	0.130
Second-generation cephalosporins(J01DC)	0.088	0.084	0.085	− 0.003	0.124	0.111
Third-generation cephalosporins (J01DD)	0.110	0.114	0.138	0.028	0.199	0.211
Fourth-generation cephalosporins (JO1DE)	0.042	0.047	0.045	0.003	0.064	0.055
Monobactams (J01DF)	0.000	0.000	0.000	0.000	0.001	0.001
Combinations of sulfonamides and trimethoprim, including derivatives (J01EE)	–	–	–	–	0.003	0.004
Carbapenenems (J01DH)	0.072	0.082	0.086	0.014	0.105	0.109
Macrolides (J01FA)	0.001	0.003	0.003	0.002	–	–
Lincosamides (J01FF)	0.023	0.024	0.022	0.000	0.028	0.022
Streptogramins (J01FG)	0.000	0.000	0.000	0.000	0.000	0.000
Aminoglycosides (J01GB)	0.055	0.055	0.051	− 0.005	0.061	0.052
Second-generation fluoroquinolones (J01MA)	0.020	0.025	0.025	0.004	0.018	0.029
Third-generation fluoroquinolones (J01MA)	0.008	0.007	0.006	− 0.002	0.012	0.007
Glycopeptides (J01XA)	0.021	0.021	0.020	− 0.001	0.037	0.033
Other antibacterials (J01XX)	0.012	0.014	0.015	0.003	0.019	0.022
Total	0.734	0.776	0.829	0.094	1.159	1.225

Data show defined daily doses per 1000 inhabitants per days, DID

*ATC* anatomical therapeutic chemical

^a^ Delta (Δ) values show the difference between 2013 and 2011 values

^b^ Muraki et al. [6]

**Fig. 1:**
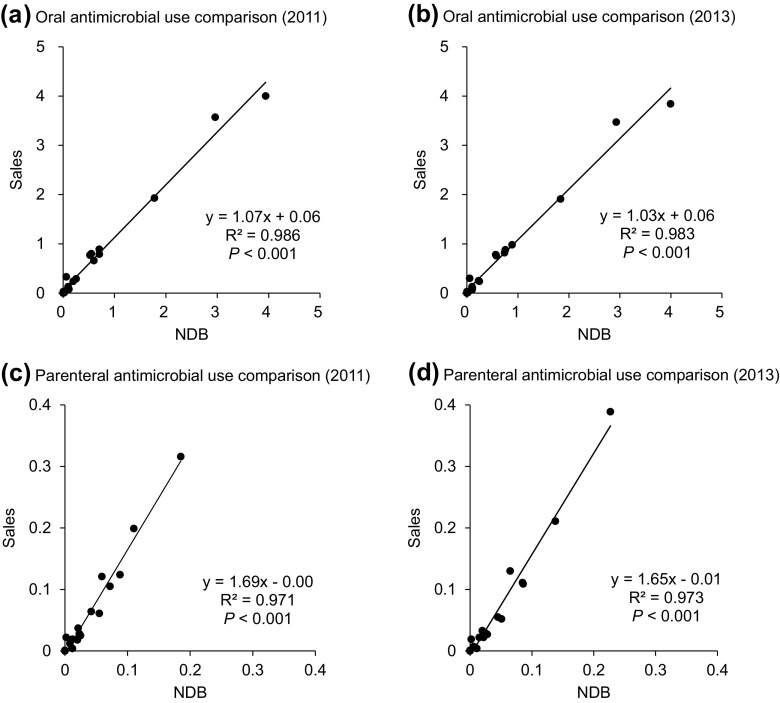
Oral and parenteral antimicrobial use comparison calculated by the NDB and the sales data in 2011 and 2013. Scatter plots represent DIDs at the ATC 4th level calculated by the NDB and sales data with linear regression. *DID* defined daily doses per 1000 inhabitants per day, *ATC* anatomical therapeutic chemical

There was a significant correlation between the DIDs at the ATC 4th level calculated by the NDB and those by sales data for both oral and parenteral antimicrobials (Fig. 1). However, the gradient of parenteral AMU showed a higher value (1.65–1.69) than that of oral AMU (1.03–1.07). This suggests that a tendency of the NDB to underestimate AMU compared to sales data, especially for parenteral antimicrobials.

**Fig. 2:**
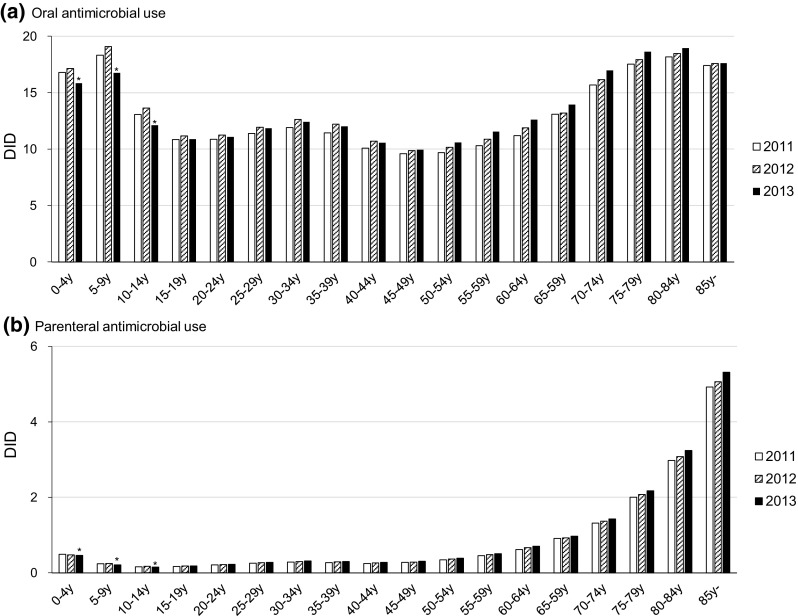
Trend of total oral and parenteral antimicrobial use stratified by age class. Asterisks indicate decreased AMU in 2013 compared with those in 2011

Total oral and parenteral AMUs stratified by age class (in 5 year increments) are shown in Fig. 2. Oral AMU stratified by age class demonstrated a U-shaped curve, with higher DIDs in the younger and older age classes. In contrast, parenteral AMU stratified by age class showed an upward-sloping curve. When stratified by age group (0–14, 15–64, 65, and above years), total oral AMU in the children group (0–14 years) was comparable to that of the elderly group (65 and above years) (Table 2). Oral AMU in the working age group (15–64 years) was approximately two-thirds of those in the other age groups (Table 2). In contrast, parenteral AMU in the elderly group was extremely high compared to the other age groups. With regard to trend of AMU, only the children group showed a decrease in both oral and parenteral AMU in 2013 compared to those in 2011 (Table 2). The percentage of the oral form of antimicrobials among the total AMU in the children, the working age, and the elderly groups during the study period were approximately 98, 97, and 88%, respectively. When comparing the AMU in the inpatient setting with those in the outpatient setting, the oral AMU was higher in the outpatient setting compared to those in the inpatient setting in all age groups. Conversely, the parenteral AMU in the inpatient setting was higher than those in the outpatient settings, especially for the elderly group (Table 2).

**Table 2:** Trends of inpatients, outpatients, and total antimicrobial use stratified by age group from 2011 to 2013

Age group	2011			2012			2013			Δ^a^
	Inpatients	Outpatients	Total	Inpatients	Outpatients	Total	Inpatients	Outpatients	Total	Total
(a) Oral										
0–14 years	0.187	15.789	15.976	0.184	16.355	16.539	0.171	14.659	14.830	− 1.147
15–64 years	0.325	10.418	10.743	0.328	10.965	11.294	0.324	11.044	11.368	0.625
65 years	1.550	14.437	15.988	1.524	14.760	16.284	1.491	15.375	16.866	0.879
(b) Parenteral										
0–14 years	0.200	0.090	0.290	0.198	0.091	0.289	0.191	0.080	0.271	− 0.019
15–64 years	0.241	0.087	0.328	0.250	0.093	0.343	0.268	0.092	0.360	0.032
65 years	1.875	0.221	2.096	2.009	0.156	2.165	2.043	0.233	2.276	0.180

Data show defined daily doses per 1000 inhabitants per days, DID

^a^ Delta (Δ) values show the difference between 2013 and 2011 values

Distribution of AMU in 2013 stratified by antimicrobial category and the three different age groups, as well as the difference between 2011 and 2013, are shown in Table 3. Regarding the oral form, third-generation cephalosphorins were the most frequently used oral antimicrobials in the children group, whereas macrolides were the most frequently used oral antimicrobials in the other age groups. Regarding the parenteral form, combinations of penicillins including β-lactamase inhibitors and cephalosporins were frequently used regardless of age group. In particular, combinations of penicillins including β-lactamase inhibitors showed a high DID value (0.738) in the elderly group. Notably, the proportion of aminoglycosides was relatively high in the children group compared to the other age groups.

**Table 3:** Antimicrobial use stratified by ATC classification and age group in 2013

ATC 4th level	Age group					
	0–14 years		15–64 years		65 years	
	DID in 2013	Δ	DID in 2013	Δ	DID in 2013	Δ
(a) Oral						
Teracycline (J01AA)	0.257 (1.7)	− 0.045	0.666 (5.9)	0.059	0.476 (2.8)	0.061
Amphenicols (J01BA)	0.000 (0.0)	0.000	0.000 (0.0)	0.000	0.000 (0.0)	0.000
Penicillins with extended spectrum (J01CA)	0.878 (5.9)	− 0.049	0.683 (6.0)	0.192	0.872 (5.2)	0.376
β-Lactamase-sensitive penicillins (J01CE)	0.087 (0.6)	− 0.012	0.013 (0.1)	− 0.001	0.009 (0.1)	− 0.002
Combinations of penicillins, including β-lactamase inhibitor (J01CR)	0.905 (6.1)	0.098	0.117 (1.0)	0.017	0.162 (1.0)	0.039
First-generation cephalosporin (J01DB)	0.142 (1.0)	− 0.021	0.084 (0.7)	− 0.008	0.136 (0.8)	− 0.010
Second-generation cephalosporins (J01DC)	0.011 (0.1)	0.000	0.063 (0.6)	− 0.006	0.062 (0.4)	− 0.007
Third-generation cephalosporins (J01DD)	5.64 (38.1)	− 0.336	2.46 (21.6)	0.047	2.71 (16.1)	− 0.06
Penems (J01DI)	0.203 (1.4)	0.019	0.089 (0.8)	0.005	0.084 (0.5)	− 0.002
Combinations of sulfonamides and trimethoprim, including derivatives (J01EE)	0.251 (1.7)	0.012	0.565 (5.0)	0.101	2.01 (11.9)	0.386
Short-acting macrolide (J01FA)	0.322 (2.2)	− 0.066	0.091 (0.8)	− 0.004	0.592 (3.5)	− 0.019
Intermediate-acting macrolide (J01FA)	4.66 (31.4)	− 0.549	3.22 (28.3)	0.084	5.57 (33.0)	0.142
Long-acting macrolide (J01FA)	0.637 (4.3)	− 0.390	0.619 (5.4)	− 0.094	0.443 (2.6)	− 0.057
Lincosamides (J 01FF)	0.003 (0.0)	0.000	0.011 (0.1)	0.001	0.012 (0.1)	0.002
First-generation fluoroquinolones (J01MA)	0.029 (0.2)	− 0.005	0.026 (0.2)	0.000	0.045 (0.3)	− 0.006
Second-generation fluoroquinolones (J01MA)	0.561 (3.8)	0.208	1.73 (15.2)	0.052	2.75 (16.3)	− 0.095
Third-generation fluoroquinolones (0J1MA)	0.029 (0.2)	− 0.001	0.848 (7.5)	0.170	0.836 (5.0)	0.134
Polymyxins (J01XB)	0.007 (0.0)	0.000	0.003 (0.0)	0.000	0.008 (0.0)	− 0.001
Other antibacterials (J01XX)	0.205 (1.4)	− 0.011	0.083 (0.7)	0.007	0.087 (0.5)	− 0.001
(b) Parenteral						
Teracycline (J01AA)	0.003 (1.2)	− 0.004	0.004 (1.1)	− 0.001	0.031 (1.4)	− 0.003
Amphenicols (J01BA)	0.000 (0.0)	0.000	0.000 (0.0)	0.000	0.000 (0.0)	0.000
Penicillins with extended spectrum (J01CA)	0.034 (12.5)	0.002	0.015 (4.3)	0.002	0.057 (2.5)	0.005
β-Lactamase-sensitive penicillins (J01CE)	0.000 (0.0)	0.000	0.001 (0.4)	0.000	0.005 (0.2)	0.001
Combinations of penicillins, including β-lactamase inhibitor (J01CR)	0.047 (17.3)	− 0.004	0.058 (16.0)	0.010	0.738 (32.4)	0.103
First-generation cephalosporins (J01DB)	0.014 (5.2)	0.001	0.040 (11.2)	0.004	0.154 (6.8)	0.009
Second-generation cephalosporins (J01DC)	0.024 (8.7)	− 0.007	0.054 (15.1)	− 0.002	0.190 (8.4)	− 0.016
Third-generation cephalosporins (J01DD)	0.055 (20.3)	− 0.001	0.063 (17.4)	0.011	0.367 (16.1)	0.067
Fourth-generation cephalosporins (JO1DE)	0.007 (2.4)	0.001	0.021 (5.7)	0.001	0.127 (5.6)	0.004
Monobactams (J01DF)	0.000 (0.0)	0.000	0.000 (0.0)	0.000	0.001 (0.1)	0.000
Carbapenenems (J01DH)	0.014 (5.1)	0.000	0.032 (9.0)	0.006	0.255 (11.2)	0.025
Short-acting macrolide (J01FA)	0.001 (0.2)	0.000	0.000 (0.0)	0.000	0.001 (0.1)	− 0.001
Long-acting macrolide (J01FA)	0.000 (0.1)	0.000	0.001 (0.3)	0.001	0.008 (0.3)	0.008
Lincosamides (J01FF)	0.010 (3.8)	− 0.006	0.014 (3.8)	0.001	0.050 (2.2)	− 0.004
Streptogramins (J01FG)	0.000 (0.0)	0.000	0.000 (0.0)	0.000	0.000 (0.0)	0.000
Aminoglycosides (J01GB)	0.052 (19.3)	− 0.004	0.029 (8.1)	− 0.003	0.102 (4.5)	− 0.017
Second-generation fluoroquinolones (J01MA)	0.000 (0.1)	0.000	0.008 (2.2)	0.001	0.078 (3.4)	0.010
Third-generation fluoroquinolones (J1MA)	0.000 (0.0)	0.000	0.002 (0.4)	− 0.001	0.020 (0.9)	− 0.007
Glycopeptides (J01XA)	0.006 (2.1)	0.001	0.009 (2.5)	0.000	0.056 (2.5)	− 0.007
Other antibacterials (J01XX)	0.004 (1.6)	0.000	0.009 (2.4)	0.001	0.036 (1.6)	0.006

*ATC* anatomical therapeutic chemical

DID in 2013 data shows defined daily doses per 1000 inhabitants per days (% among age group)

Delta (Δ) values show the difference between 2013 and 2011 value

## Discussion

This study evaluated the quantities and patterns of nation-wide AMU in Japan and its age-specific distribution using the NDB based on health insurance claims data. AMUs at the ATC 4th level measured by the NDB were correlated with those by the Sales data. During the period from 2011 to 2013, AMU in the children group (0–14 years) showed a decreasing trend, while the other age groups demonstrated an increase in AMU. AMUs stratified by antimicrobial category and age group were measured by the NDB, demonstrating that it can be used as a tool to monitor the outcome indices for the AMR action plan.

Measurement of AMU is important for combating AMR at the national level, and is also an effective measure of antimicrobial stewardship programs at the hospital level. However, there is no gold standard method for measuring AMU especially at the national level as the source of data is affected by the national health care system. There are several sources for AMU measurement: (a) sales: sales of medicinal products from wholesaler; (b) dispensed: medicines dispensed to patients at the pharmacies, either prescribed or not prescribed; (c) prescribed: prescription medicines dispensed at the pharmacies, not including over-the-counter medicines; and (d) reimbursed: medicines reimbursed by the health authorities; these medicines must be prescribed by a healthcare professional, dispensed at a pharmacy, and reimbursed by the healthcare provider [12]. In Europe, 31 administrative nation-wide AMU databases were identified. Eleven provided wholesalers’ sales data, 11 on reimbursed, 5 on prescribed, and 4 on dispensing medicines [12]. In the United States, oral AMU was measured using the IMS health Xponent database [13]. In Korea, AMU surveillance was performed using National Health Insurance claims data through the Health Insurance Review and Assessment service [14, 15].

In Japan, nation-wide AMU was previously measured by sales data [6]. In this study, we compared reimbursed data in the NDB with sales data. The DID values calculated by the NDB were almost identical (slightly underestimated) to those calculated by sales data for oral AMU (Fig. 1). Although there was some discrepancy between the data on the parenteral form of AMU, a significant linear correlation was observed. This discrepancy could be due to dead stocks in the medical institution and disposed expired medications, leading to an overestimation of sales data on the parenteral form of AMU. Importantly, the features of the NDB must be addressed. Japan has established nation-wide medical insurance, and antimicrobials are not available as the over-the-counter medication. As such, virtually, all antimicrobials were prescribed by physicians at medical institutions and reimbursed using an electrical claim system. The NDB was constructed using electronic claim data that excluded approximately 2% of claim data (non-electrical data) and specific data not covered by public health insurance such as antimicrobial prescription in nursing homes and workers’ compensation insurance. These factors could have resulted in an underestimation of AMU. However, these excluded data formed a considerably small proportion of entire AMU. As such, the NDB was considered a reliable data source for AMU surveillance presuming true usage, and can thus be an alternative to sales data.

The NDB has several advantages over the sales data in terms of patient information. Age-specific distribution of AMU as well as trend analysis was performed in this study. Oral and parenteral AMUs demonstrated different age-specific distribution. With regard to the oral form, AMUs in the children group and the elderly group showed higher AMUs than the working age group (Fig. 2 and Table 2). However, the AMU in the working age group was higher than predicted given the low morbidity of infectious diseases expected in this population. Oral antimicrobial stewardship would be more important in this group, because the percentage of the oral form of AMU out of the total AMU was high (97%) and some of them were related to antimicrobial misuse or abuse for non-bacterial infections such as the common cold [16].

With regard to the parenteral form, AMU in the elderly group demonstrated an extremely high value compared to the other age groups (Fig. 2 and Table 2). The percentage of parenteral form among total AMU was low with 6% on the whole, but that of the elderly group was relatively high at 12%. The frequency of admission was higher in the elderly group for exacerbation of infectious diseases such as pneumonia and urinary tract infection. Parenteral antimicrobials were mostly used in the inpatient setting, resulting in the high use of parenteral antibiotics among the elderly. As Japan is approaching an ageing society, promoting parenteral antimicrobial stewardship in the elderly group is of much importance.

The Japanese AMR action plan set the outcome indices to (a) reduce the AMU (DID) in 2020 to two-thirds of the level in 2013, (b) reduce the oral cephalosporins, quinolones, and macrolides DID in 2020 by 50% from the level in 2013, and (c) reduce parenteral AMU (DID) in 2020 by 20% from the level in 2013 [4]. Although both total oral and parenteral AMU increased during the period from 2011 to 2013, a decreasing trend was observed in the children group regardless of dosage form (Tables 1, 2), which might be related to the practice of antimicrobial stewardship among pediatricians. With regard to three major oral antimicrobials (cephalosporins, quinolones, and macrolides), both the DIDs of cephalosporin and macrolides showed a decreasing trend (Table 1). In contrast, DIDs of fluoroquinolones increased especially among the working age and elderly group for the third-generation fluoroquinolones (Tables 1, 3). Regarding the parenteral form, combinations of penicillins including β-lactamase inhibitors demonstrated high DIDs at baseline (in 2011) and increased DIDs in 2013 especially for the elderly group (Tables 1, 3). Therefore, assessment of AMU trend stratified by antimicrobial category and age group would be essential for promoting and monitoring AMR action.

This study has several limitations that must be addressed. Since the DDD value was not corrected for renal function and body weight, the volumes of the relevant antimicrobials expressed in DDDs were underestimated in a patient with diminished renal function and in a pediatric patient [17, 18]. The AMUs in the children group, especially in the younger age groups such as 0–4 and 5–9 years in Fig. 2, would be higher values if body weight correction was performed. To overcome this limitation, a further study using a dose-independent measure such as the number of the days of therapy should be conducted. In this study, we analyzed AMU stratified by antimicrobial category and age group. In addition to these data, the NDB Japan has more detailed information regarding gender, geographic location, and prescribing practitioner (Medical Doctor and Doctor of Dental Surgery). The database we used in this study did not include antimicrobials prescribed in the dental department of the hospital and dental clinic, although this should account for a small amount. A further study including more information is warranted.

In conclusion, there was a significant linear correlation between the AMUs measured by the sales data and the NDB, and the trend of AMU stratified by antimicrobial category and age group was successfully assessed using the NDB. As a universal health system with electronic claiming systems has been established in Japan, the NDB based on health insurance claims data has the potential to reflect real AMU in clinical practice, and can thus be a useful tool to monitor outcome indices for the AMR action plan.
